# Impact of an in-built monitoring system on family planning performance in rural Bangladesh

**DOI:** 10.1186/1478-4491-5-16

**Published:** 2007-06-07

**Authors:** Humayun Kabir, Rukhsana Gazi, Ali Ashraf, Nirod Chandra Saha

**Affiliations:** 1 Health Systems and Infectious Diseases Division, International Centre for Diarrhoeal Disease Research, Bangladesh, Dhaka, Bangladesh

## Abstract

**Background:**

During 1982–1992, the Maternal and Child Health Family Planning (MCH-FP) Extension Project (Rural) of International Centre for Diarrhoeal Disease Research, Bangladesh (ICDDR,B), in partnership with the Ministry of Health and Family Welfare (MoHFW) of the Government of Bangladesh (GoB), implemented a series of interventions in Sirajganj Sadar sub-district of Sirajganj district. These interventions were aimed at improving the planning mechanisms and for reviewing the problem-solving processes to build an effective monitoring system of the interventions at the local level of the overall system of the MOHFW, GoB.

**Methods:**

The interventions included development and testing of innovative solutions in service-delivery, provision of door-step injectables, and strengthening of the management information system (MIS). The impact of an in-built monitoring system on the overall performance was assessed during the period from June 1995 to December 1996, after the withdrawal of the interventions in 1992.

**Results:**

The results of the assessment showed that Family Welfare Assistants (FWAs) increased household-visits within the last two months, and there was a higher use of service-delivery points even after the withdrawal of the interventions. The results of the cluster surveys, conducted in 1996, showed that the selected indicators of health and family-planning services were higher than those reported by the Bangladesh Demographic and Health Survey (BDHS) 1996–1997. During June 1995-December, 1996, the contraceptive prevalence rate (CPR) increased by 13 percentage points (i.e. from 40% to 53%). Compared to the national CPR (49%), this increase was statistically significant (p < 0.05).

**Conclusion:**

The in-built monitoring systems, including effective MIS, accompanied by rapid assessments and review of performance by the programme managers, have potentials to improve family planning performance in low-performing areas.

## Background

Inadequate basic management skill among health teams at the implementation level is one of the main constraints in providing primary healthcare (PHC) in developing countries [1]. Literature on health reforms also emphasizes strengthening the capacity of the ministry of health at the central and district levels and improvement of supervision and administrative leadership [2-7]. An effective monitoring and tracking mechanism enables identification of low-reach catchments areas, operational problems in improving coverage, and corrective actions to enhance service-use [8]. In the Philippines, focus is placed on improving maternal and child health and meeting the reproductive intentions of women by improving the national management information system (MIS), making better use of existing data from various sources to produce an annual status report for the family-planning programme, and strengthening the monitoring systems at the local level [9]. There is a need to increase efficiency, decentralize the decision making process, and train health staff in the areas of management, policy, and planning [10] to implement a minimum package of cost-effective public-health measures and clinical interventions aiming at improving health conditions in low-income countries.

Pathfinder International, a Rural Service Delivery Partnership (RSDP), was a part of the National Integrated Population and Health Programme (NIPHP) of the MoHFW, GoB. The RSDP collaborated with the University of North Carolina (UNC) at Chapel Hill, United States of America (USA), to introduce a local-level monitoring system through an action-plan intervention for strengthening team work and developing the competence of health and family-planning managers and frontline supervisors at the levels of sub-district and below. The RSDP complemented the government efforts to increase the accessibility and use of the MCH-FP programme by rural families in the context of the NIPHP [11]. The action-plan intervention revealed that both number of acceptors of contraceptive methods and use of child immunization services increased, and evidence of MCH-FP performance-related meetings held at the sub-district and union levels was more systematic during the implementation of action plans [12]. The MoHFW considered the participation of stakeholders and users of health services in all phases of project cycle, (i.e. planning, implementation, monitoring, and evaluation) a vital element for achieving the goal of the Health and Population Sector Programme (HPSP). The MoHFW introduced a stakeholders committee in 1999 to develop local plans for comprehensive health and family-planning services. Systematic holding of meetings of the stakeholders committee carried positive effects in improving the delivery of health and family-planning services, while the meetings also ensured the monitoring of performance of local health facilities [13].

In Bangladesh, the delivery of health and family planning services for 300 000 rural populations is coordinated from the sub-district (upazila), the lowest administrative structure with substantial responsibilities for planning and implementation of all development activities in rural areas [14]. The Directorate of Family Planning (DFP) administers doorstep delivery of the family planning programme, particularly in rural areas, by its female grassroots workers, known as Family Welfare Assistants (FWAs). The FWA visits the home of each married woman of reproductive age (MWRA) once every two months to provide information and counselling on family planning, distribute oral pills and condoms, disseminate information about the services available at the various service centres, and refer clients to service centres. The MIS Unit of the DFP was established in 1979 to meet the information needs of both family planning and maternal and child health [15]. Rajshahi division has experienced the highest contraceptive use-rate in the country since 1983, followed by Khulna division [16]. However, Sirajganj was a low-performing sub-district located in the highest performing division. In 1983, the CPR in Sirajganj was only 8%, while the national CPR for rural areas was about 19%. There was a sharp decline in the total fertility rate (TFR) at Sirajganj from 6.4 in 1983–1985 to about 3.8 in 1990–1992. The CPR stabilized at about 40% during 1990–1995. The desired family size in Sirajganj was over 3.0 in 1993 and has declined slightly since then [17]. Lack of population based information has traditionally been one of the key drawbacks to formulating timely, responsive health policies in much of the developing world. In usual situation, the administrator or policy-maker requests data from an information or evaluation unit, which, in turn, presents either an analysis of existing data or conducts a field survey [18].

The MCH-FP Extension Project (Rural), in collaboration with the MoHFW introduced a local-level monitoring system during 1982–1992 in Sirajganj for improving the management capability where the programme managers had reviewed the progress of the performance of service providers on a few selected indicators from monthly service statistics using the MIS in various meetings. The FWA Register was designed as a longitudinal record keeping system for the FWA. Under the leadership of MIS Unit of the DFP, it provided a foundation for the monitoring of FWA activities through supervisory field-visits [19]. Fortnightly meetings, mid-level supervisory meetings, and salary-day meetings were held once a month among local-level managers, service providers, and supervisors to review the performance of the programme and to identify the problems and barriers. Development of a strategy plan to address those barriers was a component of the monitoring system. This provided a venue for review of performance, identification, and solution of problems. This study was designed to assess the overall performance despite the operational changes that have since occurred. Since Sirajganj was a low-performing area in terms of indicators on health and family-planning, the present study was intended to evaluate whether an in-built monitoring system and local level planning would improve the performance.

## Materials and methods

The study was conducted to assess the impact of an in-built monitoring system on the sustainability of a few selected indicators of MCH-FP after the withdrawal of the interventions.

A cross-sectional study design was followed. Both quantitative and qualitative methods were used for collecting data on selected indicators. A rapid survey methodology was developed to provide administrators with quick information on problems faced at the community level. The cluster sampling procedure has been used throughout the world in immunization surveys. The EPI (Expanded Programme on Immunization) 30-cluster rapid assessment survey was used as the quantitative method for collecting data on selected indicators. Multi-stage, simple random sampling was used – one in July 1995 and the other one in December 1996 – in order to minimize the sample size required. A list of villages of all unions (one sub-district consist 8–10 unions having average population of 25 000–30 000) was used as a sampling frame. Twenty villages were selected, covering all the unions of the sub-district. Selection of the number of villages from each union was proportional to the size of the union. A cluster of 30 MWRA from each village was selected that yielded a sample of 600 MWRA for interview. Female interviewers received seven days' intensive training on data collection using various research methods and techniques. They interviewed 600 MWRA under the supervision of a field research officer who had more than seven years' experience in field research work and had supervisory and monitoring skills. The interviewer asked the responsible person of the sample village to select a primary school or mosque/temple/church/pagoda (a place of worship). One household from one specific corner of the worship place or primary school was selected as an index household. The corner was specified beforehand and was constant for all the selected villages. Interviews of neighbouring permanent residence MWRA, following the one in the index household, continued until interviews of 30 such MWRA were completed. Female respondents were selected because they were the major recipients of reproductive healthcare services. The major indicators of health and family-planning were: (a) awareness about services available from FWAs; (b) frequency of contacts with FWAs; (c) number of desired children; (d) unmet contraceptive need; (e) accessibility to H&FWCs and SCs; and (f) use of contraceptive methods. Design effect was used for establishing that 210 children (i.e. 30 clusters with 7 children per cluster) are necessary for a survey. In this study, the required sample was doubled to avoid the design effect.

The qualitative methods were used for assessing the routine activities of 66 FWAs. The routine activities observed included: (a) administration of injectables at the door-step and (b) record-keeping. Two research officers made three days' observations on the activities of each FWA in a year during their home- visits. A structured observation checklist was used. At the facility level, three categories of meetings were observed, namely:

(i) fortnightly meetings of FWAs and their immediate supervisor and paramedics at H&FWC to review the performance of the previous month and current stock of contraceptives;

(ii) monthly mid-level supervisory meetings and local managers to review the union-wise monthly performance, discuss the field problems, and made decision to solve those problems; and

(iii) monthly salary-day meetings of field and union-level service providers, supervisors, and local managers to review the MCH-FP-related performance of field workers and paramedics. A structured observation checklist was used for collecting information on 34 meeting proceedings. Content analysis of meeting minutes was done, and reports from observers of meetings were analyzed manually.

Univariate analysis was conducted using SPSS (version 10) to determine different indicators of health and family-planning use. Chi-square test was employed to observe any significant differences in proportions between the first cluster survey (referent) and the second cluster survey.

### Limitation

In absence of division-wise selected indicators, we used the national survey data of BDHS 1996–1997 to compare the selected indicators of health and family planning service use with the cluster survey or rapid assessment survey.

## Results

### Observation of routine activities of FWAs

Data of 1995 and 1996 showed a consistent pattern of adherence to the recommended protocol for administration of injectables (Table 1). The skills of the FWAs remained very high (99%), and the FWAs followed the procedures necessary for the maintenance of correct-recording in the FWA Register. However, the FWAs did not strictly follow the checklists for screening the pill and injectable contraceptive users.

**Table 1:** Observation of routine activities of Family Welfare Assistants (FWAs)

Activity	1995 (n = 66)	1996 (n = 66)
	
	**%**correct	**% **correct
Proportion of FWA who filled in the register	96	99
Proportion of FWA who used the checklists for client screening	50	50
Proportion of FWA who followed the recommended protocol during administering injectable contraceptive	98	99

### Performance review through meetings

All the 3 categories of meetings – salary day, mid-level supervisory, and H&FWC meetings were – monitored through an observation checklist. Thirty-four meetings in 1995 and 36 meetings in 1996 were observed. All types of meetings were regularly held, although in some cases there was a delay of one-and-a-half hours or two hours in starting the meetings. Each meeting continued for about 2–3 hours in general, and attendance was satisfactory (90–95%). Other than salary day meetings, formalities in terms of the recording of the agenda and post-discussion resolutions of issues after discussion were maintained in the majority of the meetings. The monthly performance of maternal child health and family planning indicators were reviewed in the majority (70%) of the meetings.

### Client surveys

**Figure 1:**
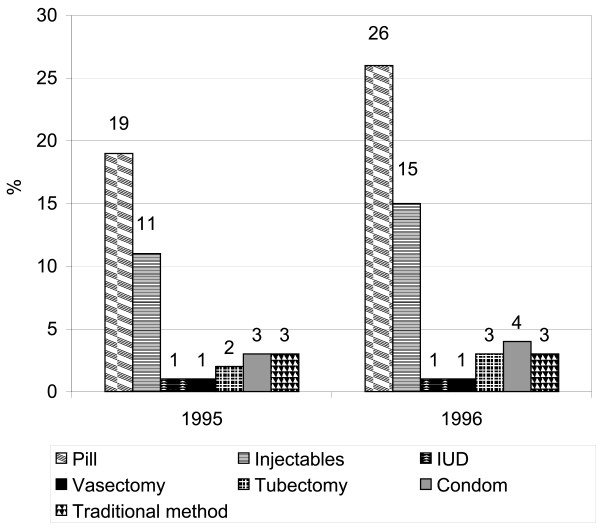
Method-specific contraceptive prevalence rate, by year, in Sirajgonj.

Table 2 shows a 13% increase in the CPR in Sirajganj (from 40% in 1995 to 53% at the end of 1996) over a 18-month period (odds ratio ([OR] = 0.59; 95% confidence interval [CI] 0.47–0.73). The difference was significant (p < 0.05). Fecund women, who were neither pregnant nor amenorrheic and who were not using any family-planning method, expressed their desire to wait for two or more years to be pregnant again, which may be considered an unmet need for family planning. The unmet contraceptive need declined from 30% in 1995 to 21% in 1996 (OR = 1.6; CI 1.25–2.06). However, desire for no additional children remained the same in 1995 and 1996 (OR = 1.0; CI 0.80–1.24). There was an indication of greater accessibility to contraceptive services, which was reflected in more frequent contacts between the FWAs and their clients within the last two months (OR = 0.79; CI 0.63–0.98) and a higher use of SCs and H&FWCs in 1996 than in 1995. The use of SCs and H&FWCs increased, respectively, from 14% to 29% and 34% to 42% during the period from June 1995 to December 1996 in Sirajganj (OR = 0.40; CI 0.30–0.53). The differences were statistically significant (p < 0.05). The results of the cluster surveys conducted in 1996 showed that the selected indicators of the use of health and family-planning services were higher than those reported by the BDHS 1996–1997, except the unmet contraceptive need (Table 3). The increase in the CPR was attributable to all methods, except for vasectomy, from 1995 to 1996 (Figure 1). The most noticeable changes were observed in the use of pills and injectables.

**Table 2:** Cluster survey results of selected indicators of health and family-planning service use in Sirajgonj after the withdrawal of the interventions

Indicator	After 1 year	After 2 year	Odds ratio
	(1995)	(1996)	
	(n = 648)	(n = 775)	
	%	%	(95% CI)
Couples desired no more children	58	58	1.0 (0.80–1.24)
Unmet contraceptive need	30	21	1.6 (1.25–2.06)**
Contraceptive prevalence rate	40	53	0.59 (0.47–0.73)**
MWRA who had ever			
Visited SC	14	29	0.40 (0.30–0.53)**
Visited H&FWC	34	42	0.71 (0.57–0.89)**
Received FWA visit within the last 2 months	51	57	0.79 (0.63–0.98)**

Results of 1995 (Referent)

**Statistically significant from 1 to 2 year(s) after the withdrawal of the interventions at 95% confidence interval, p < 0.05

CI = Confidence interval; FWA = Family Welfare Assistant;

H&FWC = Health and Family Welfare Centre; SC = Satellite Clinic

**Table 3:** Comparison of selected indicators of health and family-planning service use between the national and the cluster survey

	National survey	Cluster	Odds ratio
Indicator	(BDHS 1996–997)	survey	(95% CI)
	(n = 8,450)	(1996)	
	%	(n = 775)	
		%	
Couples desired no more children	49.1	58	0.70 (0.60–0.81)**
Unmet contraceptive need	16	21	0.72 (0.59–0.86)**
Contraceptive prevalence rate	49	53	0.85 (0.73–0.99)**
MWRA who had ever			
Visited SC	20	29	0.61 (0.52–0.72)**
Visited H&FWC	-	42	
Received FWA visit within the last 2 months	35	57	0.41 (0.35–0.47)**

Data of BDHS 1996–1997 (Referent)

**Statistically significant difference found compared to the BDHS 1996–1997, after the withdrawal of the interventions at 95% confidence interval, p < 0.05

CI = Confidence interval; FWA = Family Welfare Assistant;

H&FWC = Health and Family Welfare Centre; SC = Satellite Clinic;

Data source: BDHS = Bangladesh Demographic and Health Survey 1996–97

## Discussion

The remarkable improvement in programme performance as reported in the present study is attributable to two major factors: first, a series of on-the-job-training activities were conducted on the record keeping system, screening checklists of family planning methods, administering injectable contraceptive, management of side-effects of contraceptive methods, supervision and monitoring, etc, that updated the existing knowledge and facilitated close interaction between the trainers and the trainees. Maintenance of the active learning process, use of feedback mechanisms, and job related hands-on training were instrumental. The FWAs almost universally maintained the recommended protocol for administering injectables even after the withdrawal of the interventions. The high coverage of routine activities of the FWAs, such as record-keeping and screening of contraceptive methods, was also sustained after the withdrawal of the interventions.

Second, conducting regular performance review meetings was very powerful. The feedback system in those meetings was ensured to evolve close interaction between field workers and supervisors. The local manager had the opportunity to identify any problems and explore appropriate solutions. This led to efficient management of the programme and advance planning. In the meetings, high-performing workers were praised, and poor-performing workers were offered assistance. This process was found to be useful for monitoring individual performance and aggregated outputs at the union levels, which finally created positive attitude and improved motivation for the entire team. Thus, overall improvement of the programme performance took place. Another influential factor was the independent local survey, which was indicator-based and that ultimately motivated the programme managers to fix the target, organize field activities, and generally improve. The FWAs who provided services at the door step motivated their clients to avail of better-quality services at the fixed site centres. The higher use of SC and H&FWC services was an indicator of improved field activities.

The in-built review system was crucial. The district and local managers reviewed the results of the rapid assessment survey. These reviews assisted the sub-district managers and front-line supervisors in identifying the weakness of the programme and develop field operational strategies. The local managers then instructed the front-line supervisors to strengthen their monitoring and supervision activities (to improve the work of field-level workers), which were reflected in the survey results. This was an effect of the managers' motivation and positive efforts towards the improvement of the programme.

In a review paper on performance monitoring for family planning, experiences of different countries have been highlighted [9]. Indonesia has been one of the most successful developing countries to meet its demographic objectives. It has a strong management-oriented data system, which was created and maintained using a bottom-up approach. Findings of a case study in the Philippines revealed that the better use of existing data from various sources produced an annual status report for the Philippine Family Planning Programme (PFPP) and strengthened the monitoring systems at the local level. Such a performance monitoring system, thus, provides feedback to the management process itself. Findings of another case study done in Zimbabwe have shown that relatively simple MIS generated reliable and useful information complemented by special survey data.

The present study succeeded in using a package of strong MIS systems, performance review meetings having feedback mechanism, in-service training, and ad-hoc rapid assessment surveys to improve the performance of the programme, particularly in the low-performing areas of Bangladesh.
